# Supplementary material for: Novel ANO1 Inhibitor from Mallotus apelta Extract Exerts Anticancer Activity through Downregulation of ANO1
Source: Int J Mol Sci. 2020 Sep 4;21(18):6470. doi: 10.3390/ijms21186470 (PMC7576493; doi:10.3390/ijms21186470)
Supplement: Supplementary file 1 [file ijms-21-06470-s001.pdf]

SUPPLEMENTARY MATERIAL

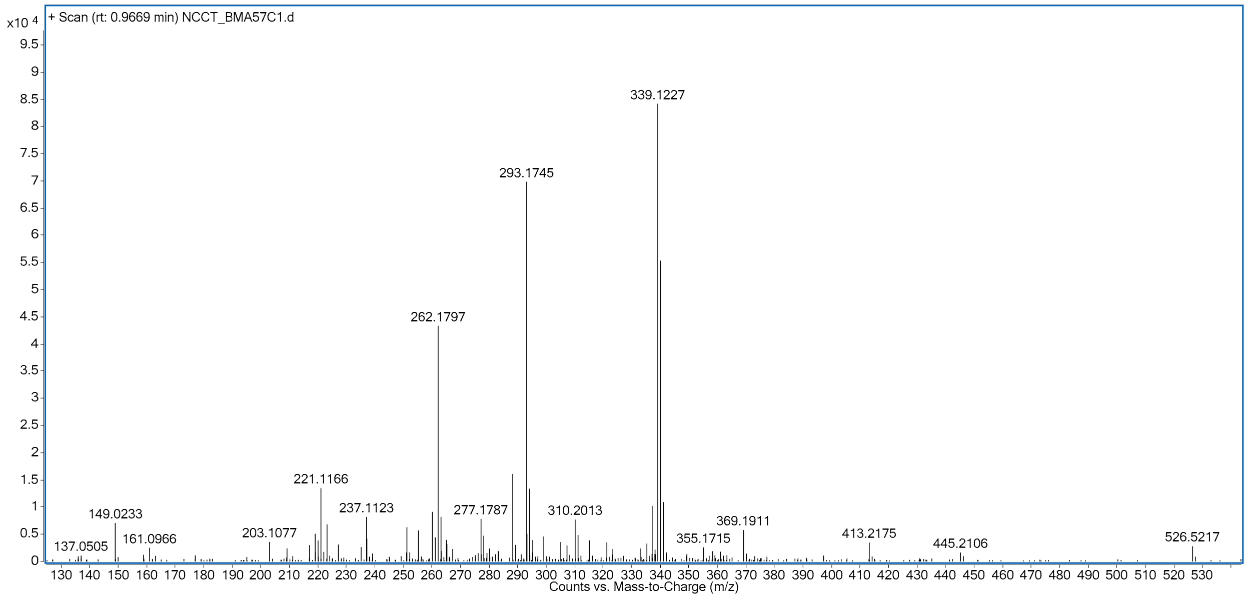

Figure S1. HR-ESI-MS of compound **1**

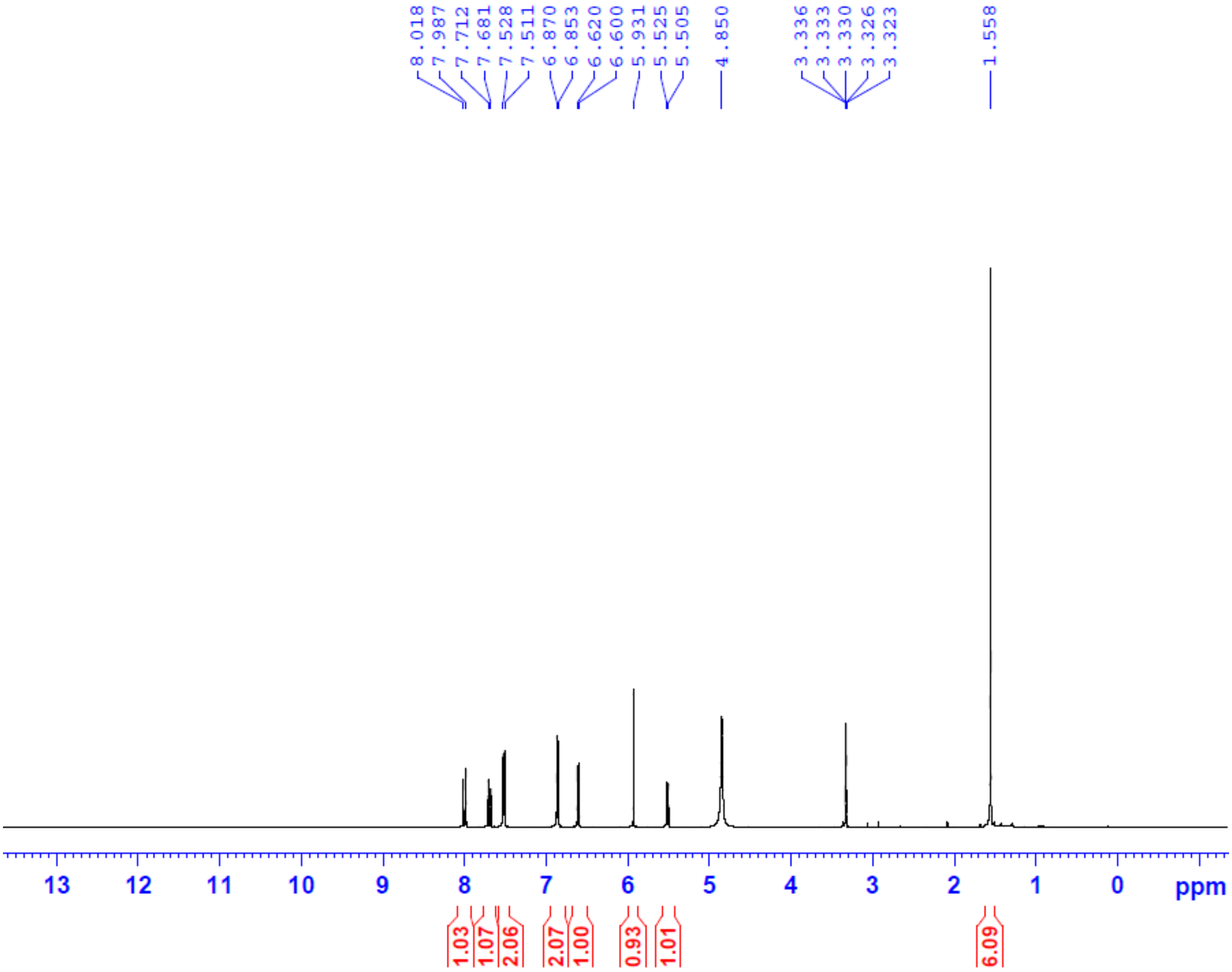

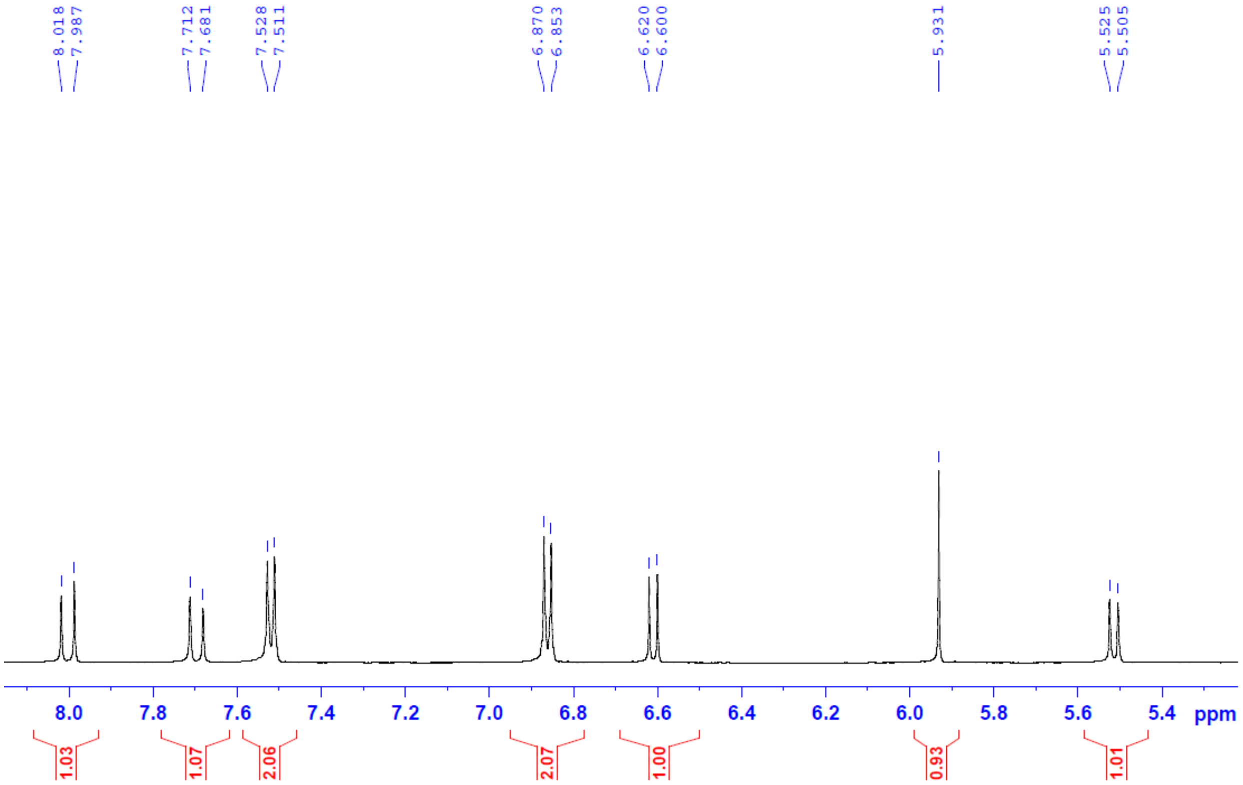

Figure S2. ¹H-NMR spectrum of compound **1**

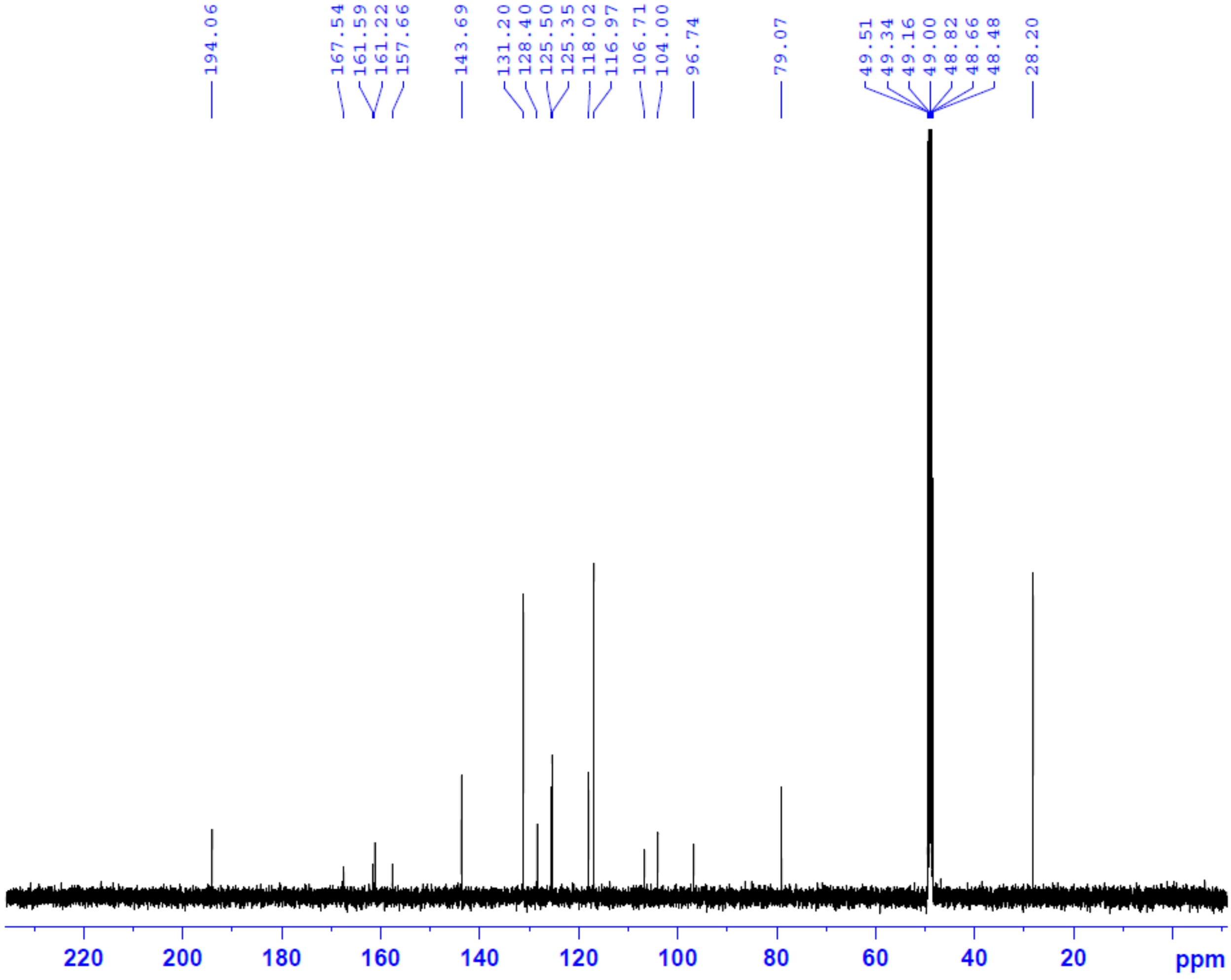

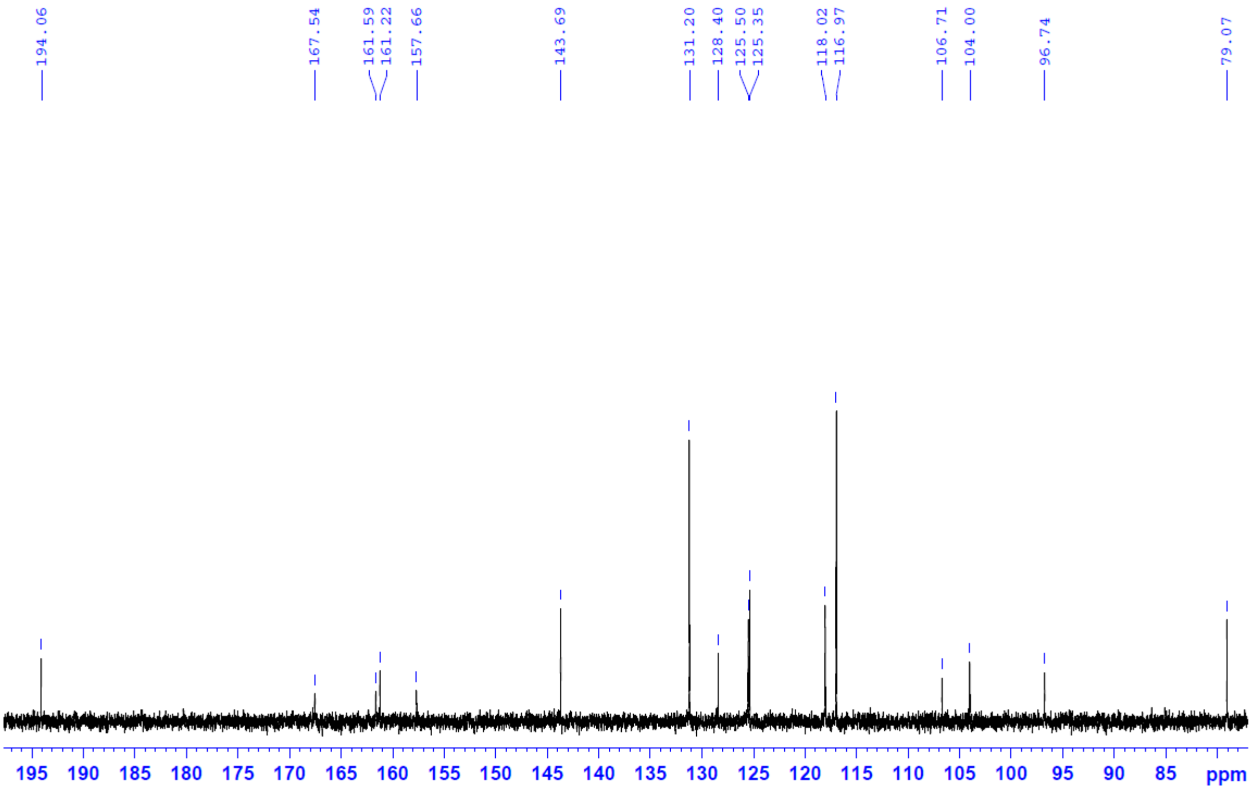

Figure S3. ^{13}C-NMR spectrum of compound **1**

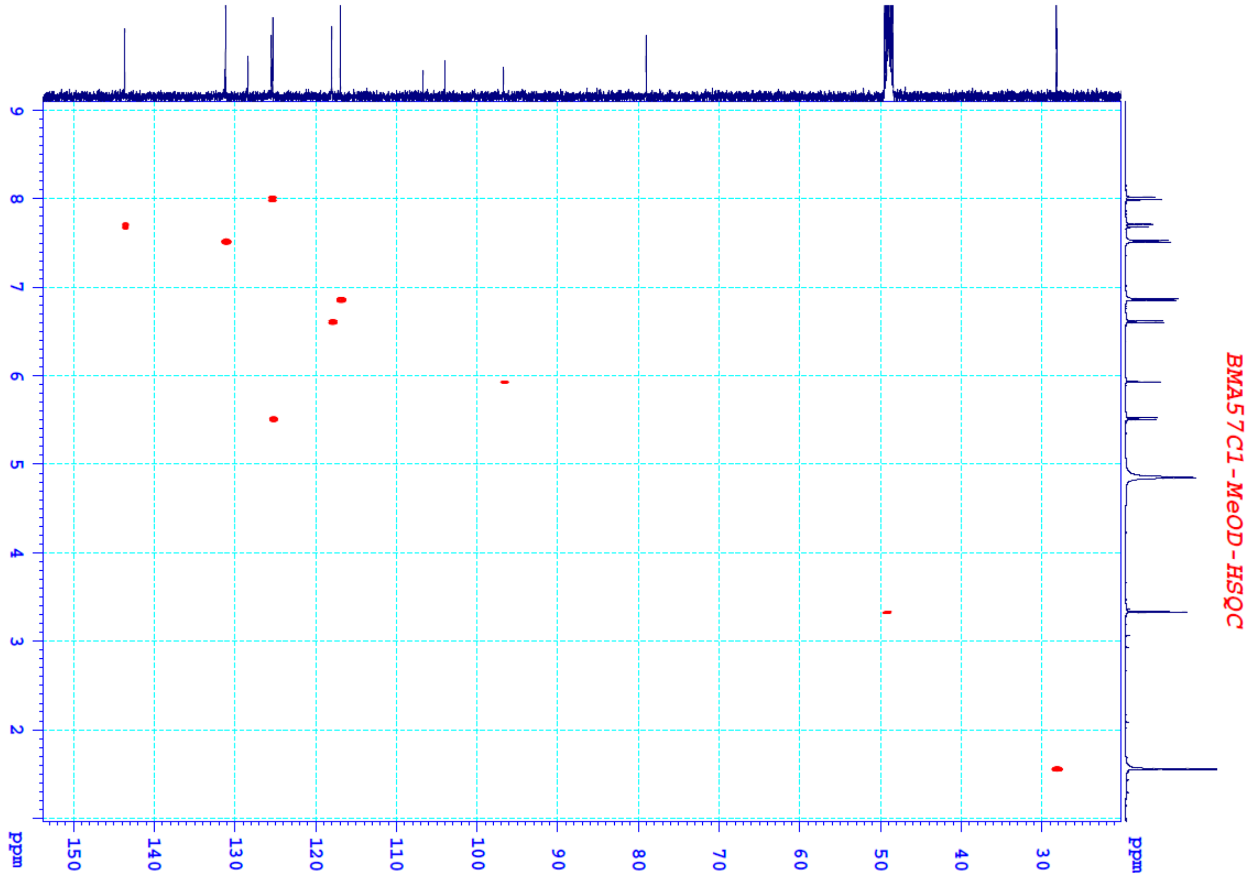

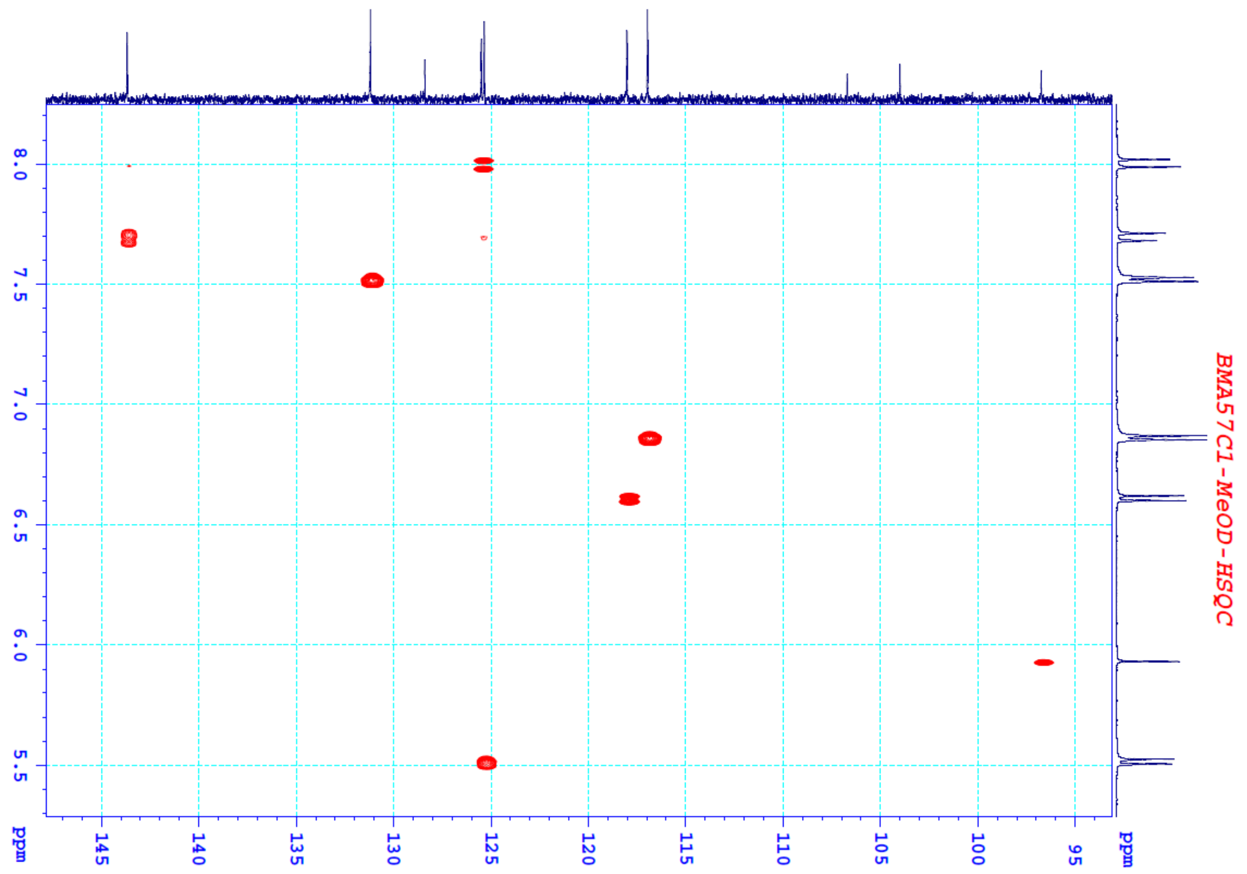

Figure S4. HSQC spectrum of compound **1**

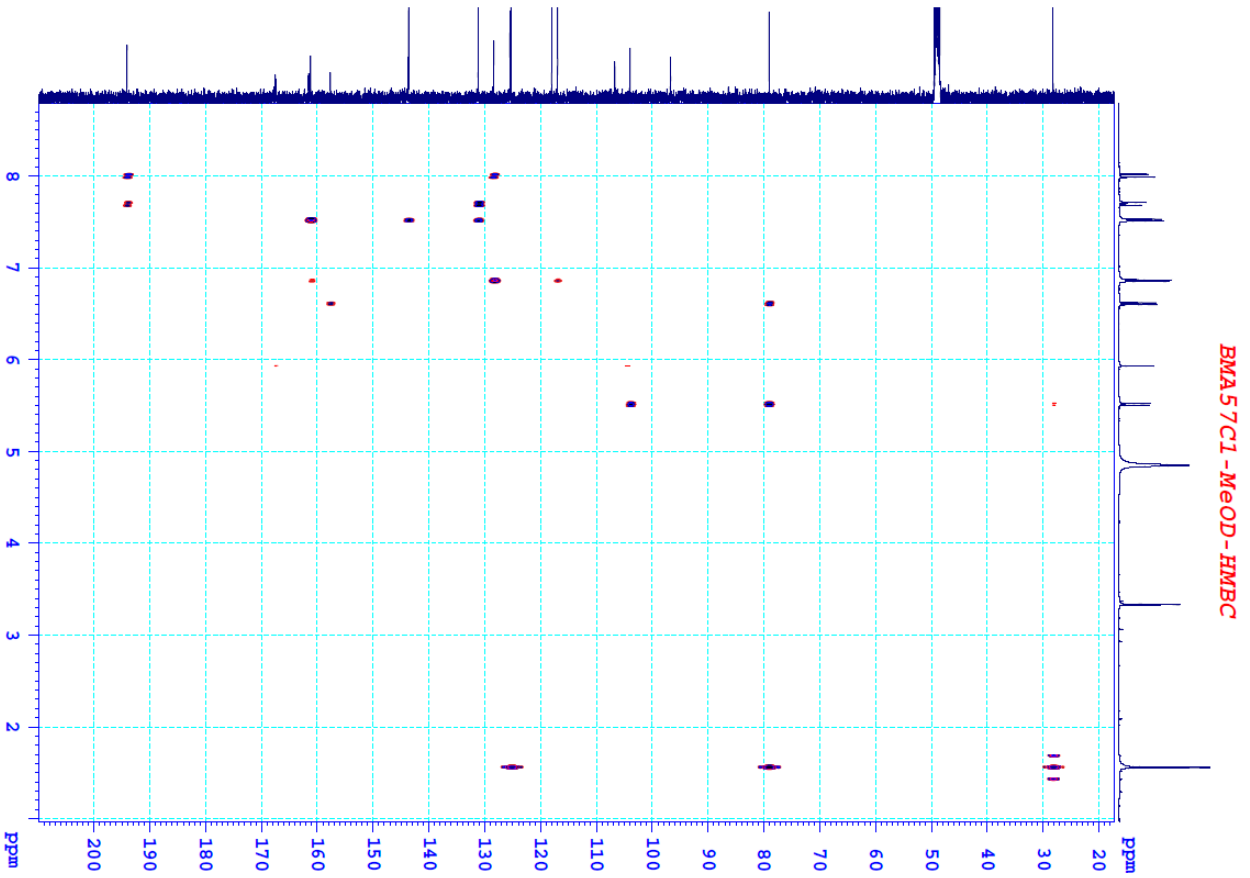

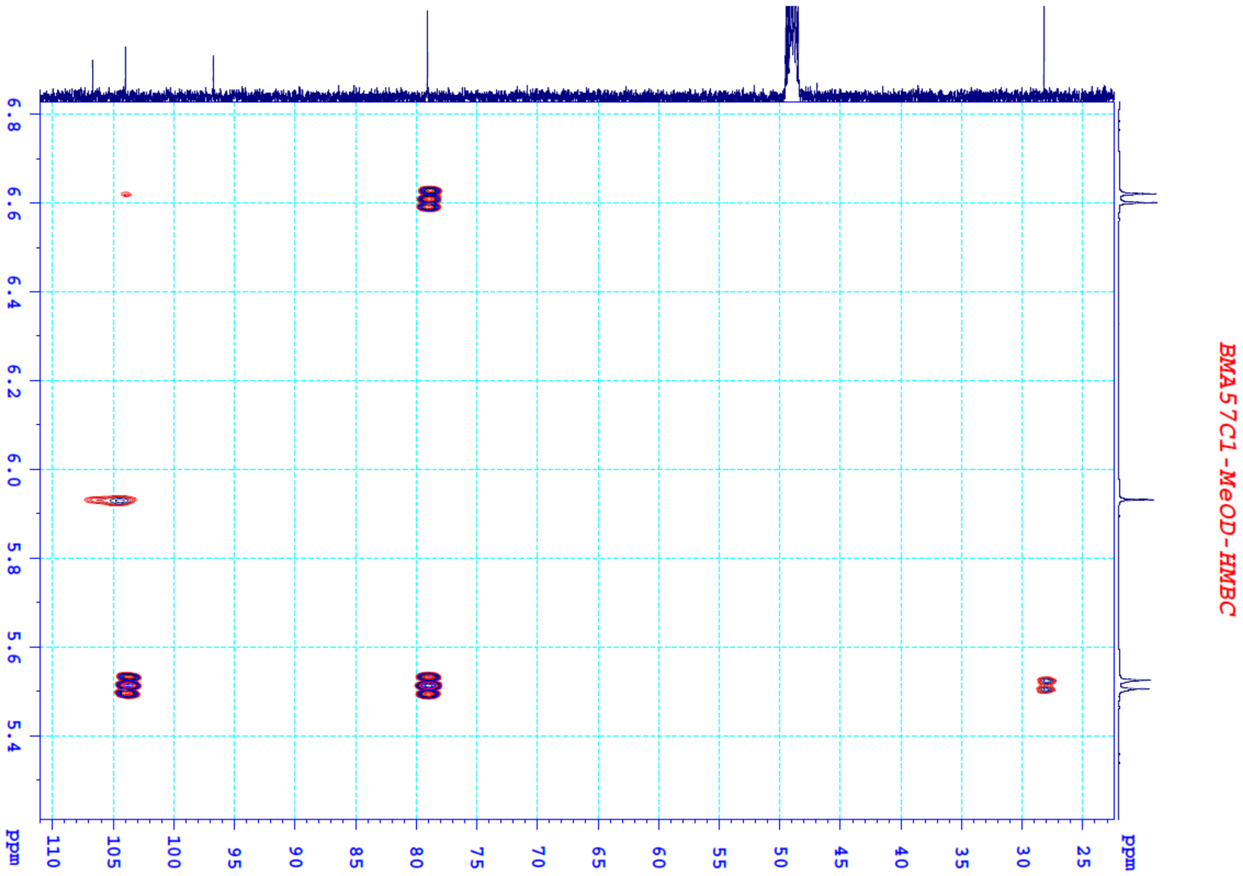

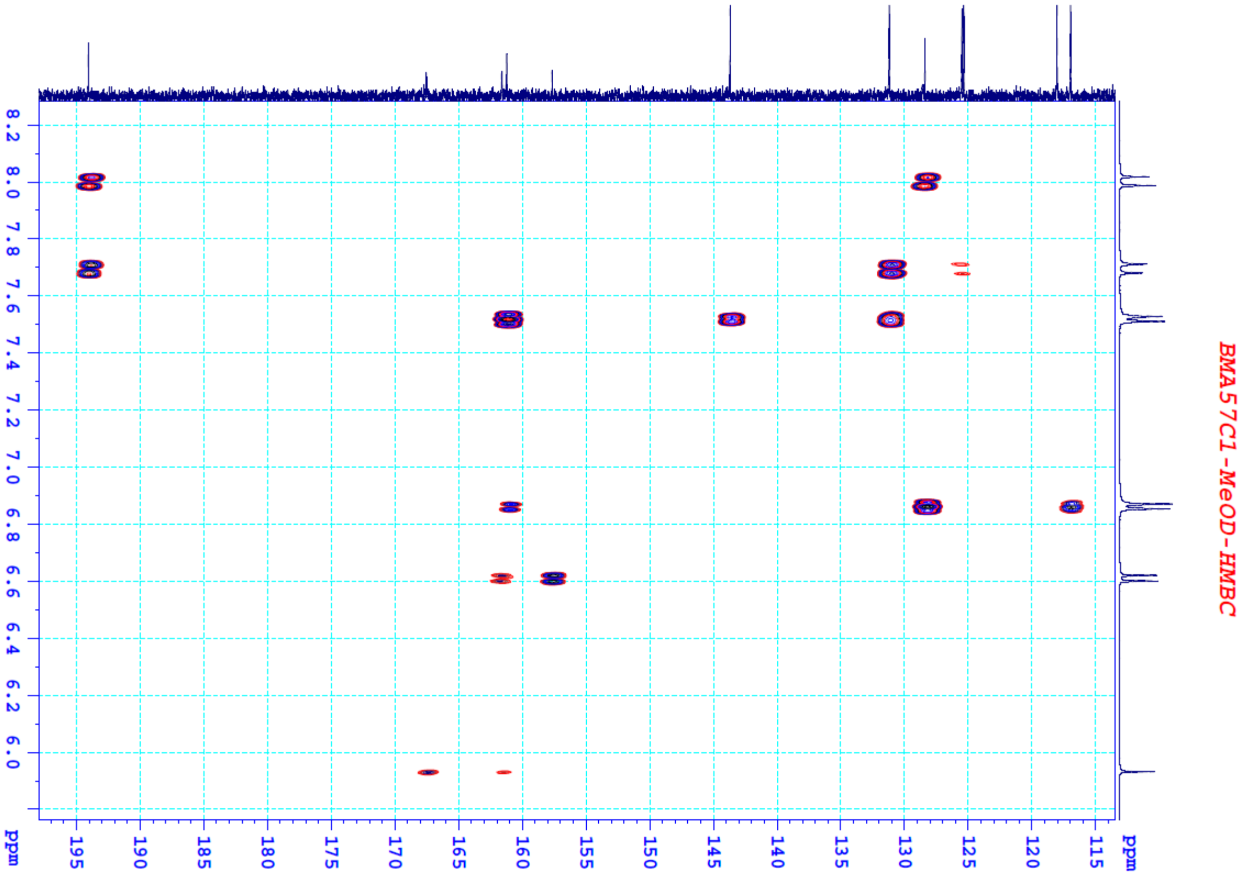

Figure S5. HMBC spectrum of compound **1**

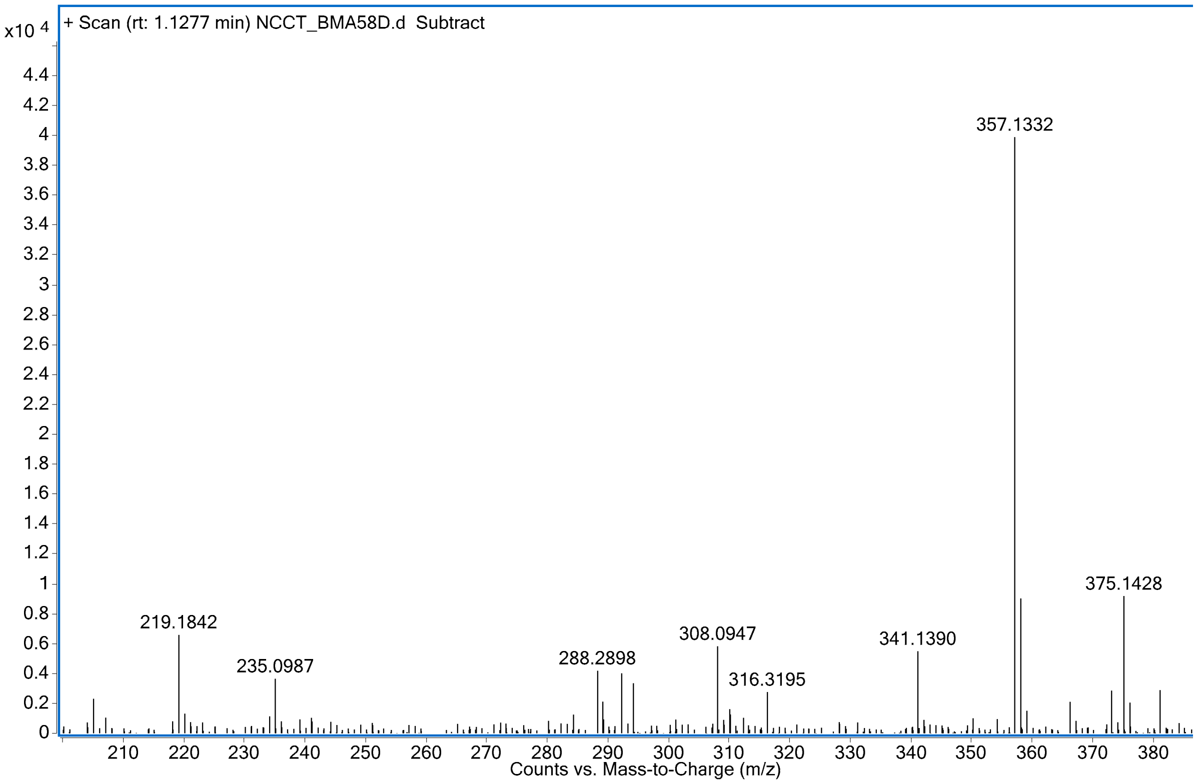

Figure S6. HR-ESI-MS of compound **2**

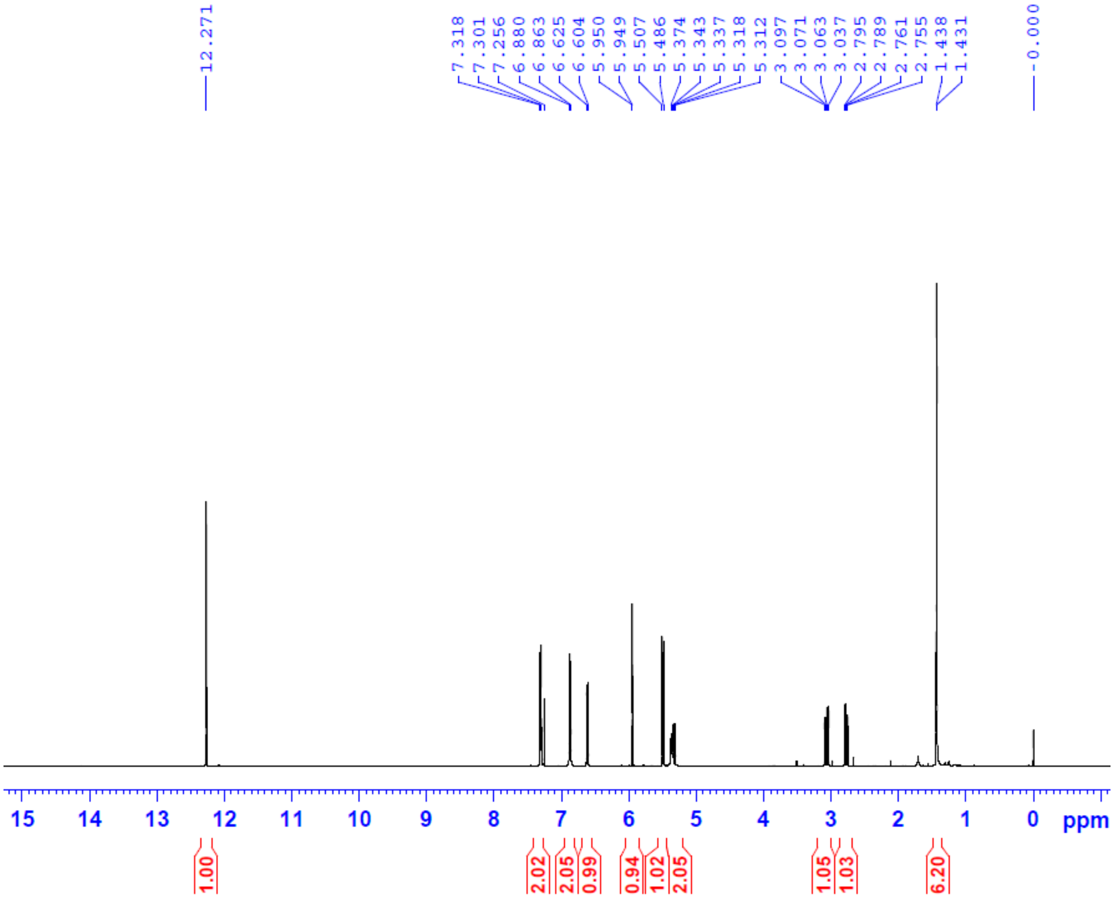

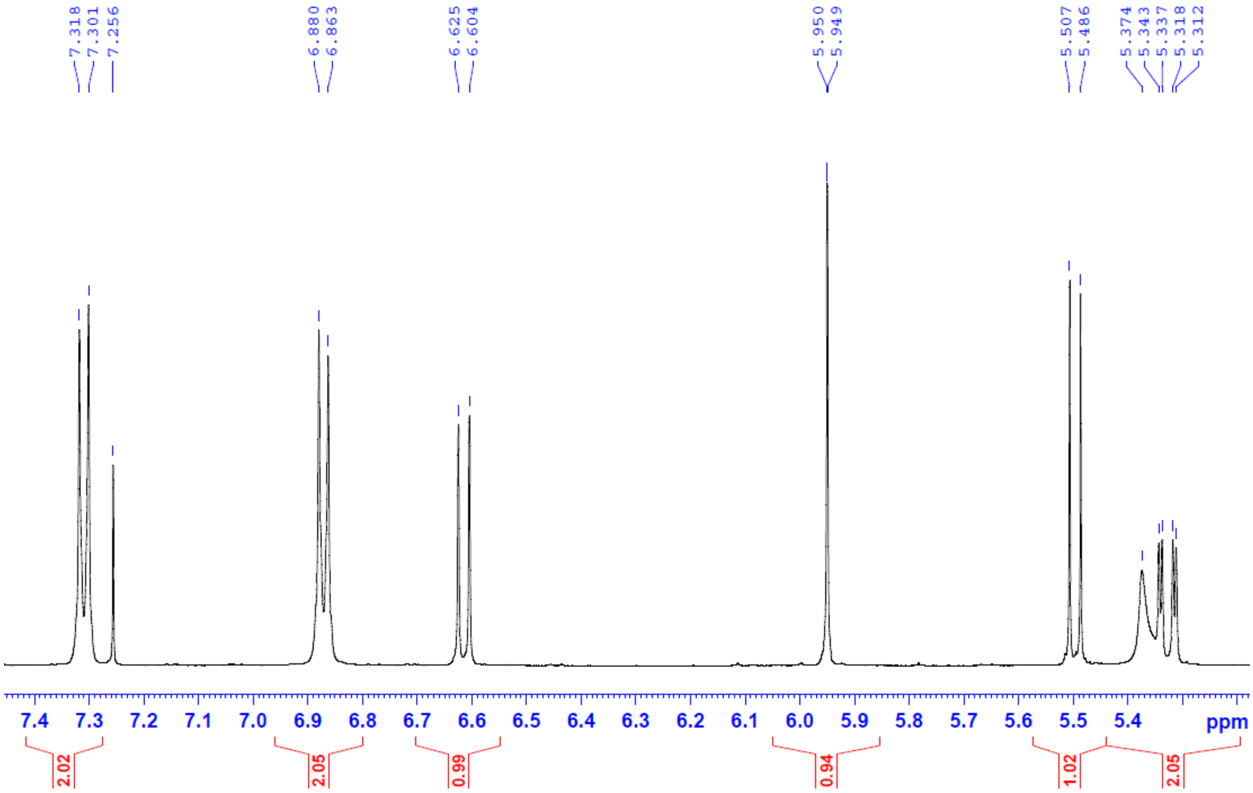

Figure S7. ^{1}H-NMR spectrum of compound **2**

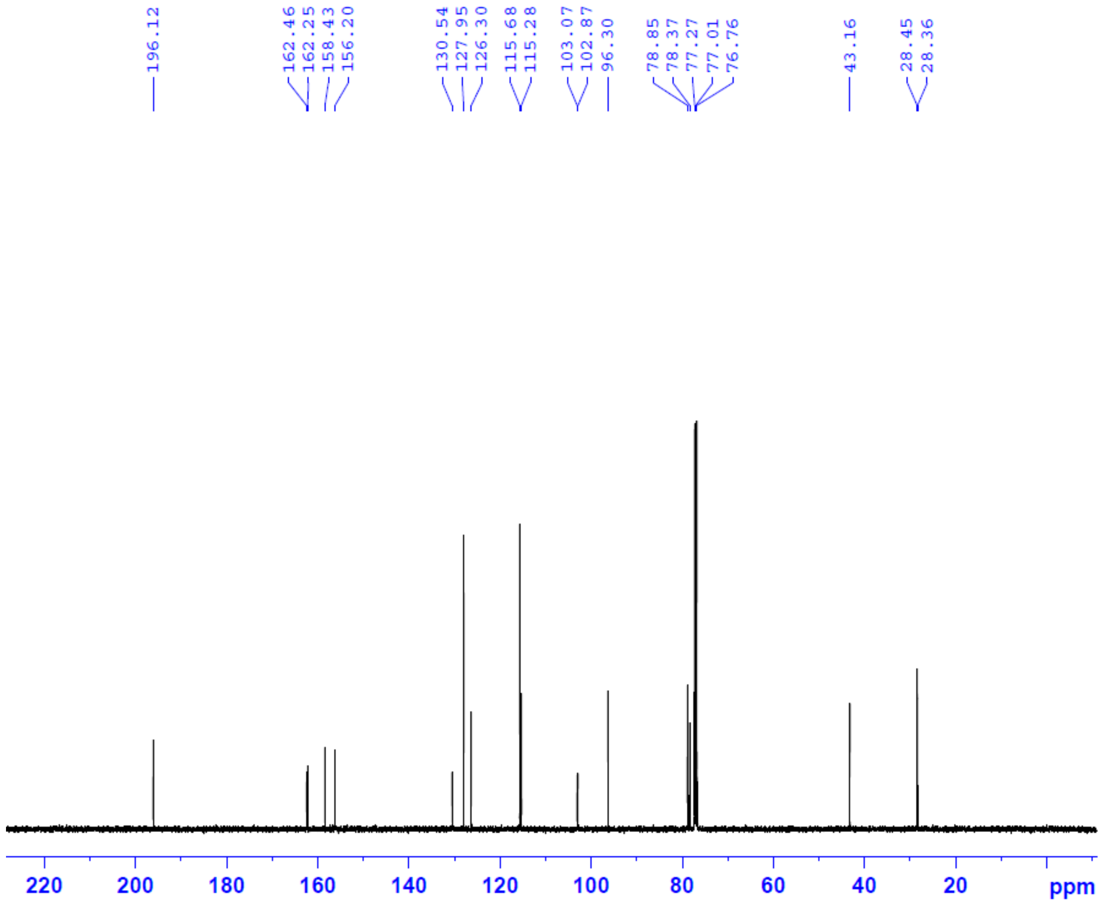

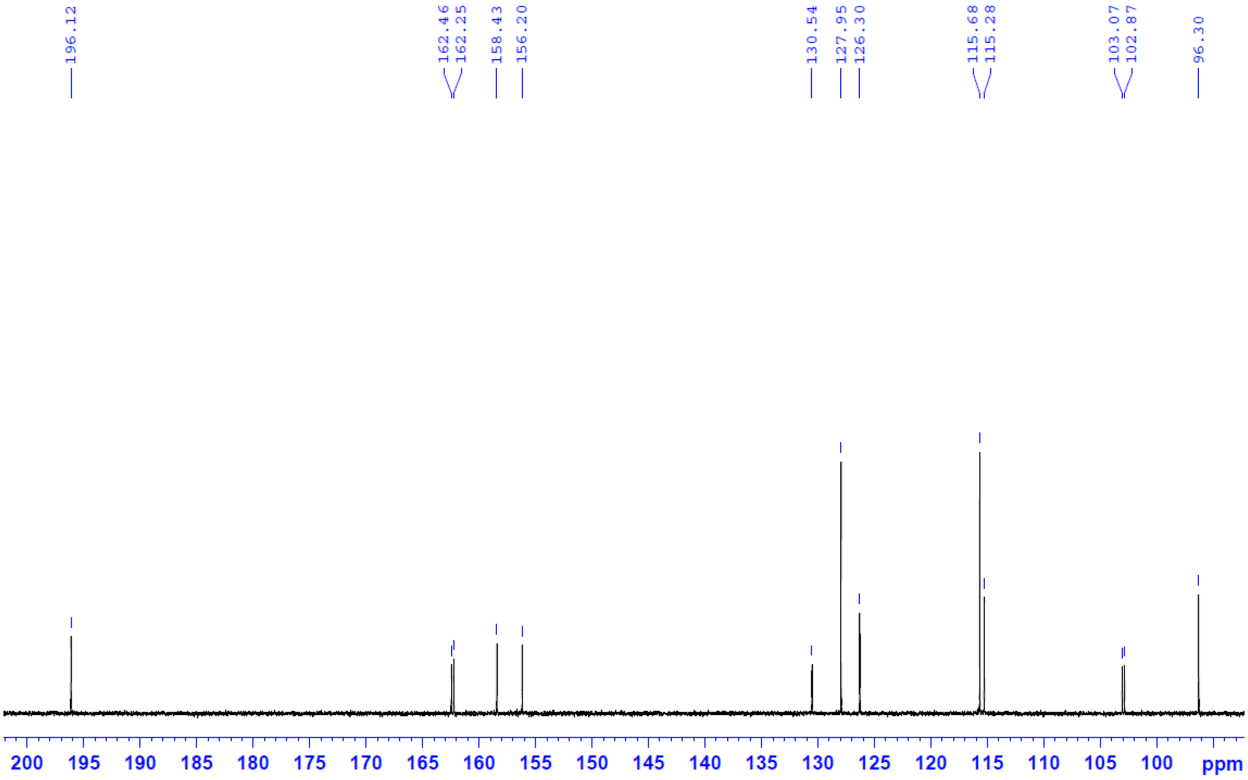

Figure S8. ^{13}C-NMR spectrum of compound **2**

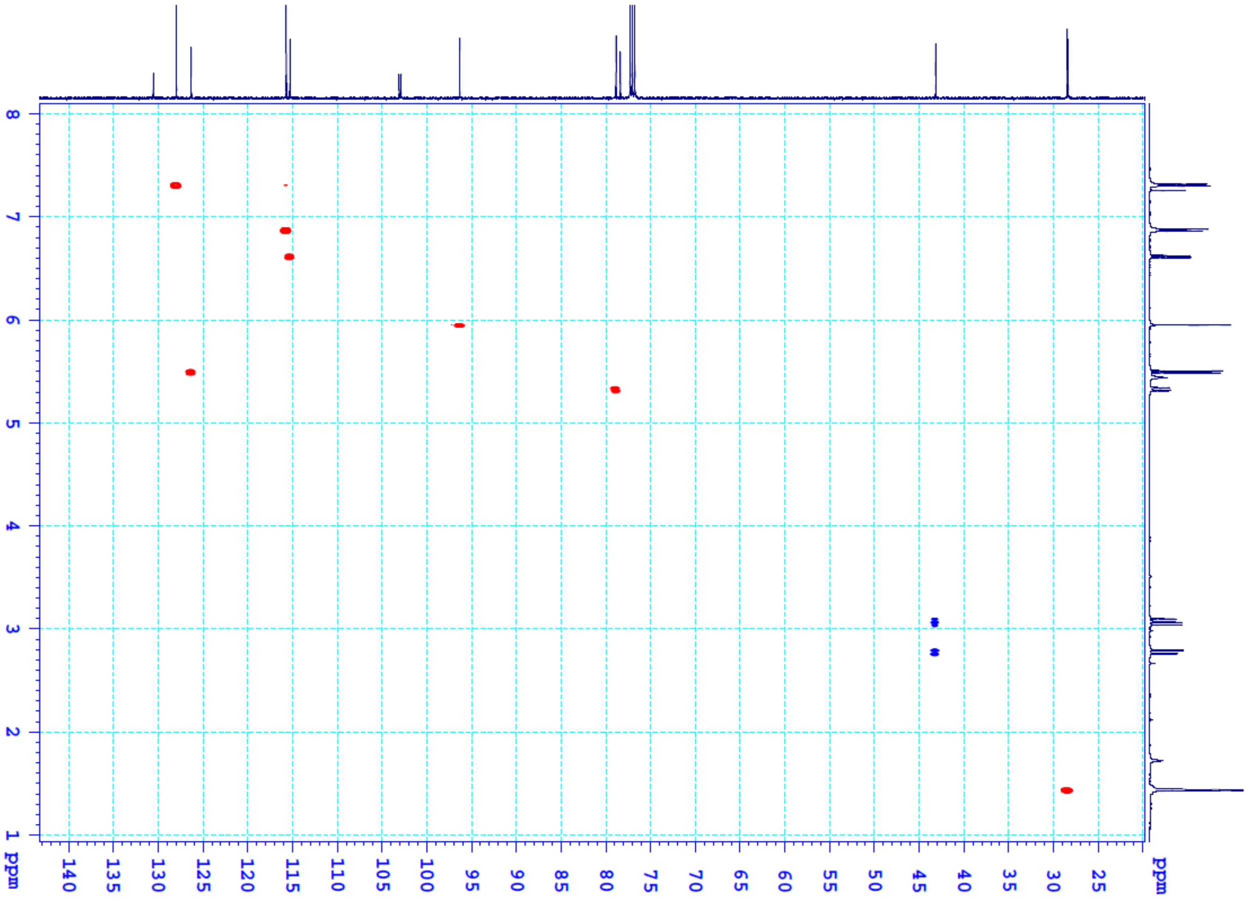

Figure S9. HSQC spectrum of compound **2**

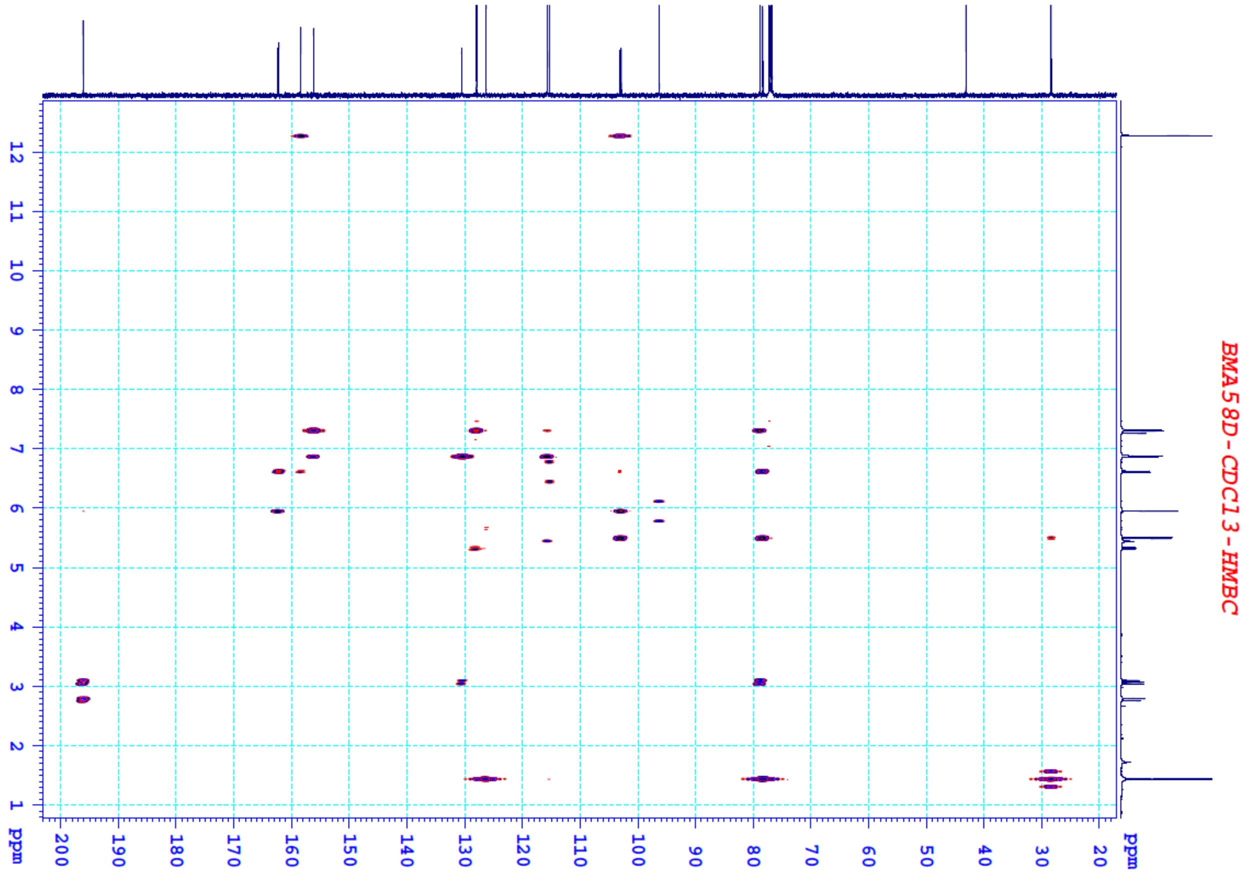

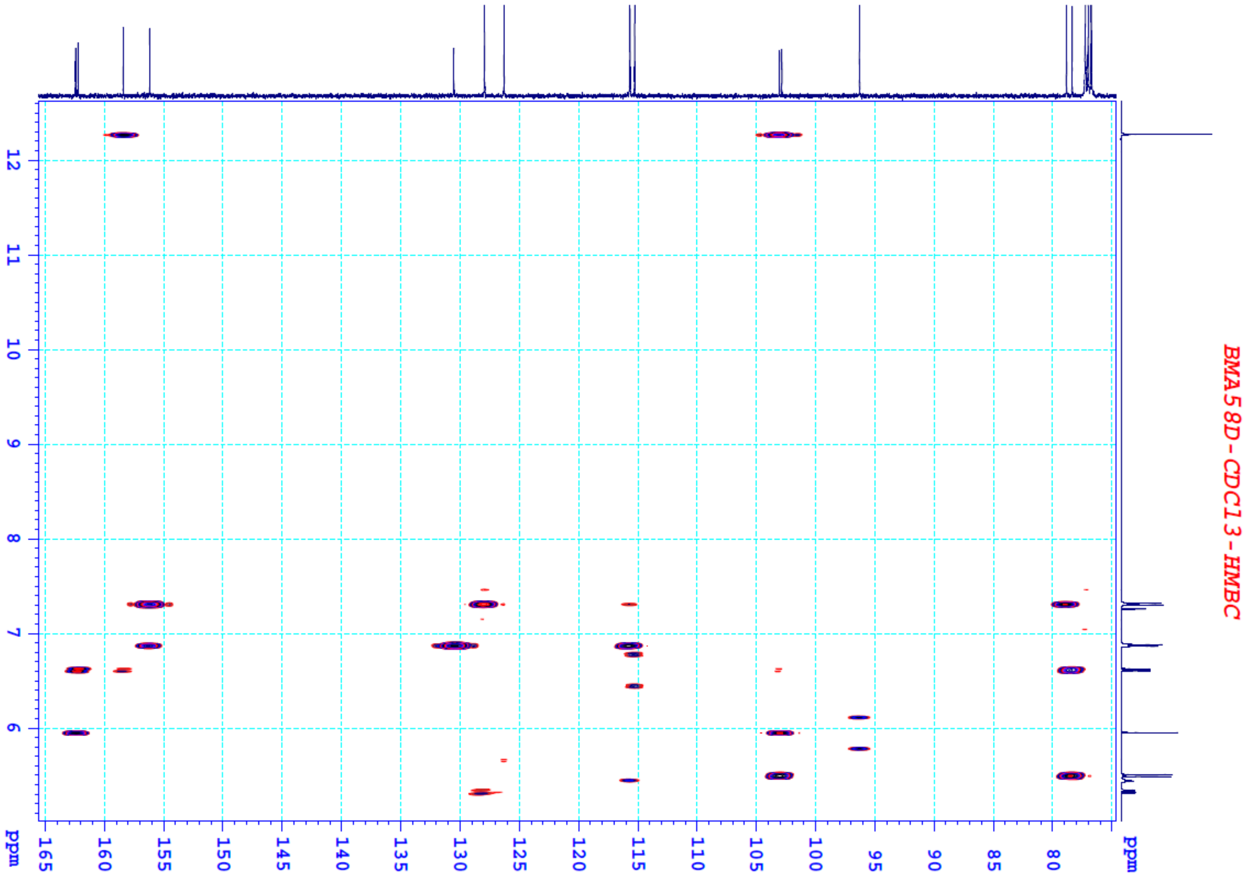

Figure S10. HMBC spectrum of compound **2**

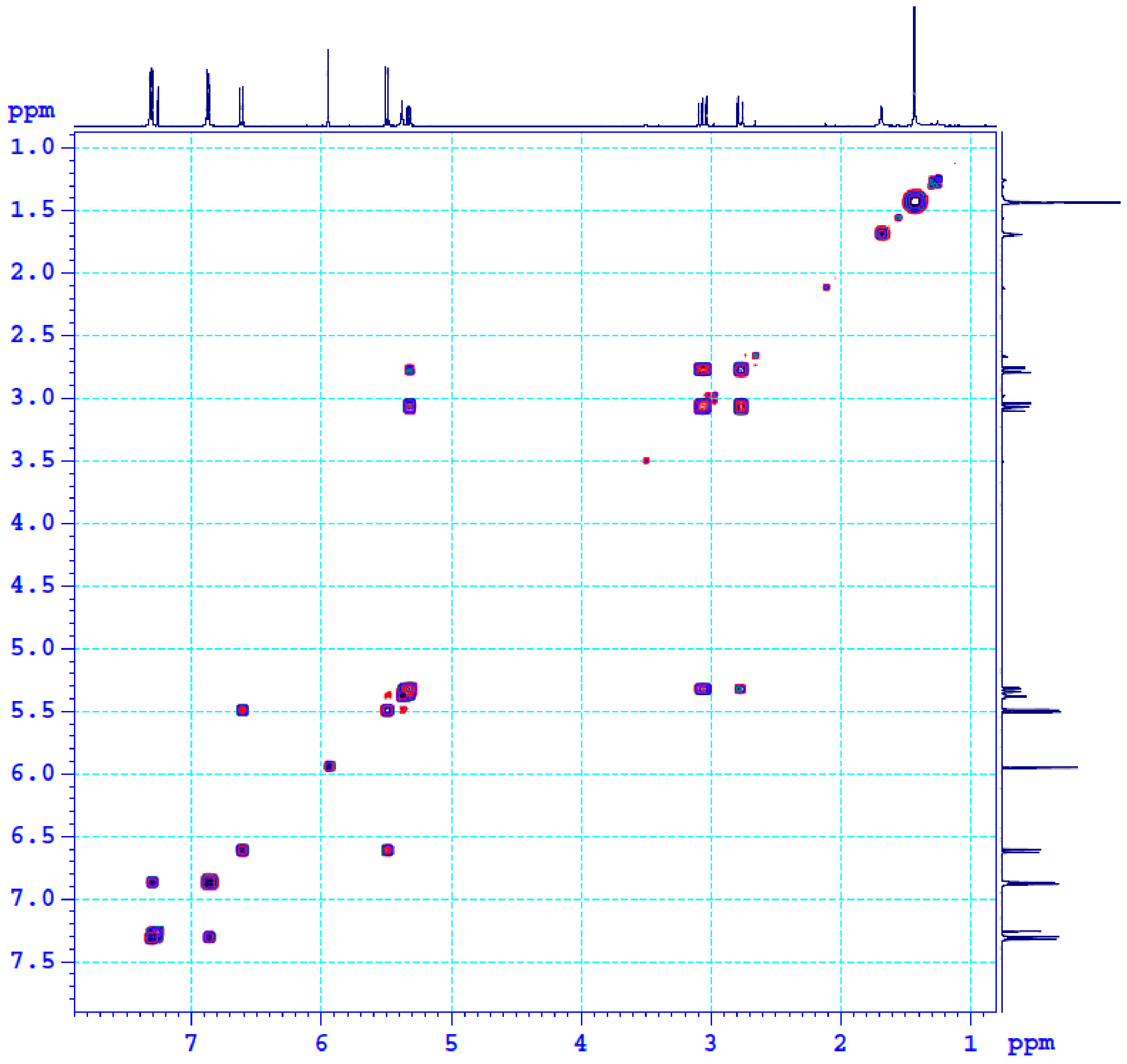

Figure S11. COSY spectrum of compound **2**

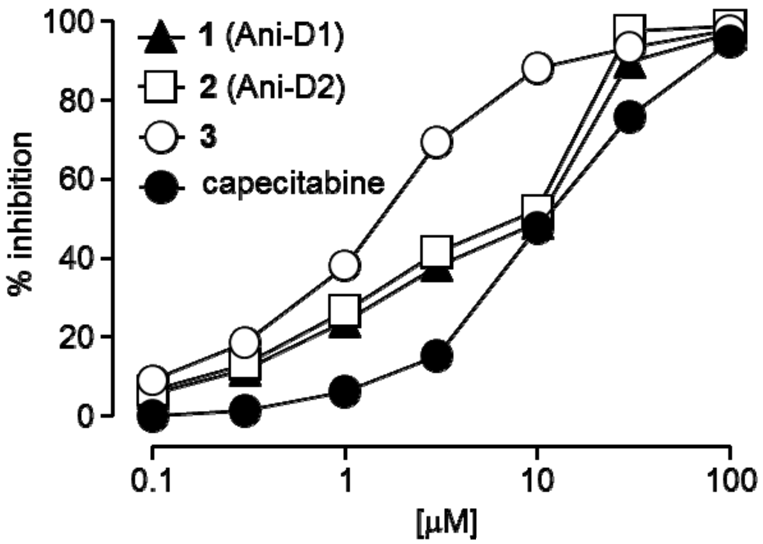

Figure S12. Dose response curve assessing the effect of compound 1-3 and capecitabine on cell viability in PC-3 cells. PC-3 cells were treated with compound 1-3 and capecitabine for 48 h, and the cell viability was determined using MTS assay kit (mean ± S.E., n = 3).

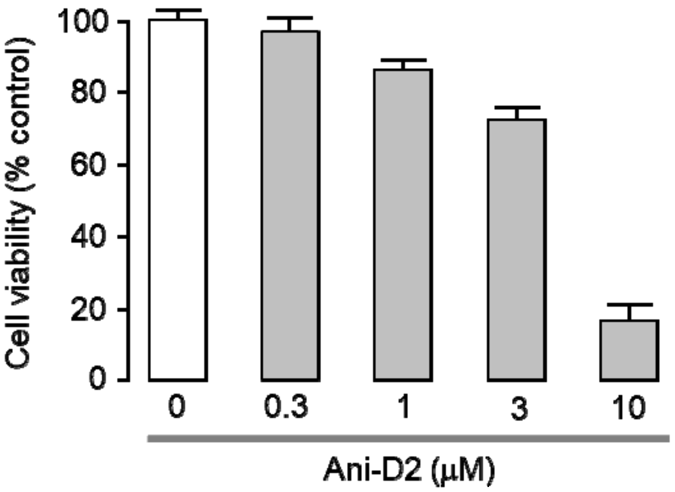

Figure S13. Effect of Ani-D2 on cell viability in HaCaT cells. Cells were treated with Ani-D2 at the indicated concentrations for 72 h, and cell viability was determined using MTS assay kit (mean ± S.E., n = 3).
